# Urban Slums: A Supportive Ecosystem for Typhoidal *Salmonellae*

**DOI:** 10.1093/infdis/jiy324

**Published:** 2018-07-27

**Authors:** Stephen P Luby

**Affiliations:** Infectious Diseases and Geographic Medicine, Stanford University, Stanford, California

**Keywords:** drinking water, poverty areas, typhoid fever, vaccines, water purification

## Abstract

The typhoidal *Salmonellae* were controlled in cities in North America and Europe in the late 19th and early 20th century by development of centralized water treatment systems. In the early 21st century, large urban centers where drinking water routinely mixes with human feces have the highest burden of typhoid fever. Although improving municipal drinking water quality is the most robust approach to reduce enteric fever burden, the high costs and managerial capacity that such systems require and decreasing per capita water availability requires new approaches to reach the highest risk communities. The spread of antimicrobial resistance threatens to increase the burden of enteric fever much sooner than the extension of safe reliable water service delivery can be implemented. Thus, vaccination is an important interim measure.

## URBAN TRANSMISSION OF THE TYPHOIDAL *SALMONELLAE*

Enteric fever, that is, bloodstream infection with *Salmonella* Typhi or *Salmonella* Paratyphi, is a life-threatening illness. In the late 19th century after cities across the United States and Europe implemented initial strategies to improve the microbiological quality of their drinking water, deaths from typhoid fever dropped immediately by a mean of 78% [1, 2]. Subsequent progressive improvements in the microbiological quality of municipal drinking water quality were associated with progressive reductions in typhoid fever incidence [3]. In some settings where untreated human feces was used to directly fertilize agricultural crops, typhoid fever transmission continued, until this transmission pathway was interrupted by the construction of wastewater treatment plants [4, 5].

In the early 21st century, the highest incidence of typhoid fever is reported from poor neighborhoods in large cities in low-income countries [6–9]. In these communities, some piped water infrastructure is commonly present, although water is usually supplied only intermittently, often for only a few hours on a few days per week [10–12]. Indeed, no major city in India provides continuous piped drinking water 24 hours per day, 7 days a week to all of its residents [13].

All piped water distribution systems leak [14]. Water utilities estimate the amount of leakage by calculating the amount of “non-revenue water”, that is, the difference between the volume of water put into a water distribution system and the volume that is billed to customers. In a database including city water utilities located in 63 countries, the median nonrevenue water loss was 29% of water supplied in 2011 [15]. Nonrevenue water combines physical system leakage with unbilled water resulting from faulty metering or corruption, but even in professionally managed systems in high-income countries, it is common for 15% of water to be lost [14, 15]. The Asian Development Bank estimates that 25% to 40% of water generated by municipal water suppliers across Asia is nonrevenue water [16].

When utilities supply water continuously, water in a leaky pipe is at higher pressure than the environment surrounding the pipe, so treated water leaks into the environment. By contrast, with an intermittent water supply, when the water is turned off, pressure drops within the pipe and material that surround the water pipe leak into the pipe [17]. In neighborhoods where feces is disposed untreated into the environment, the surrounding material that is drawn into the water pipe when the water is not running is often contaminated with the community’s fecal stream. An intermittent water supply is a contaminated water supply [18]. Indeed, this connection between a dense population’s fecal stream [19] and their water supply creates a remarkably efficient man-made pathway to transmit typhoidal *Salmonella* throughout a community [20].

Studies of the water supply in urban settings with a high incidence of typhoid fever have identified high concentrations of *S* Typhi and *S* Paratyphi in the water. In a year-long study when researchers collected weekly water samples from the 10 water sources most commonly used by communities near Patan Hospital in Kathmandu, Nepal, 77% of the drinking water samples had *S* Typhi deoxyribonucleic acid (DNA) and 70% had *S* Paratyphi DNA detectable through quantitative real-time polymerase chain reaction [21]. When multiple members of a single household in these neighborhoods were diagnosed with typhoid fever, 80% of the strains of the second infection in the household were genetically distinct from the initial household strain [22]. This suggests that new cases were arising from the diverse strains in the environment, rather than proximal person-to-person transmission. These findings emphasize the centrality of water contamination in driving the high incidence of typhoid fever in Kathmandu.

Interventions to improve water distribution can reduce the risk of typhoid. The Karnataka Urban Water Sector Improvement Project upgraded water delivery to a nearly continuous water supply for 81000 consumers in specific wards in Hubli and Dharwad, adjacent cities in Karnataka state, India [18]. Researchers compared water contamination and health outcomes among households in these intervention wards with households in nearby matched communities that continued to receive water intermittently. Seventy percent of both intervention and control households had no connection to sewers. Before the project, water ran an average 5–6 hours per day in both intervention and control areas [17]. After the intervention, households located in wards that received the continuous water supply were much less likely to have *Escherichia coli* detected in their drinking water (0.7%) compared with households in the intermittent water supply communities (31.7%) [18]. In the 3 years after the intervention, households in the continuous supply wards were 42% less likely to report at least 1 case of typhoid fever compared with households in the control wards [23].

Taken together, these data suggest that intermittent water supply in low-income cities, through pipes with numerous breaks that course through environments heavily contaminated by human feces, provides a remarkably efficient conduit for typhoidal *Salmonella* transmission.

## BARRIERS TO IMPROVING URBAN DRINKING WATER QUALITY

The best approach to reducing the burden of typhoidal *Salmonella* and other waterborne diseases would be to upgrade the supply of drinking water so that all households received safe drinking water 24 hours a day. However, there are substantial barriers to realizing this optimal solution within the next several years. Substantial funding is required to build and operate water treatment plants, pumps, pipes, and water meters and to pay the salaries of the administrative and technical personnel to operate them. Although these costs are often seen as prohibitive to extending water services to low-income communities, the poor are willing to pay for water [24]. In fact, because low- income households are so ill-served by current utilities, the poor typically end up paying 10–20 times more per liter for water than do wealthy residents of their same city [24, 25]. Providing excellent water service to low-income communities is rarely a priority for city politicians [26], especially when many of those communities are located in areas not designated for housing, and when politicians receive financial kickbacks from tanker truck operators and other informal providers of water services to these communities [24, 27–29].

Increasing populations in neighborhoods with poor water and sanitary infrastructure increases the number of people at risk for typhoid fever. Between 1990 and 2012, the number of urban slum residents in Asia increased by 100 million [30]. Although economic growth is expected to support improved infrastructure in many cities, the urban poor, especially those who live in informal settlements, are the last group who will receive high-quality water infrastructure and so will continue to be at risk for typhoidal *Salmonella* infection.

The enormous population growth of Asian cities places an additional barrier to optimal water service. Between 2015 and 2040, the population in Asian cities is projected to increase by 1 billion people [31]. These larger urban populations markedly increase demand for fresh water. Climate change is projected to further decrease the availability of fresh water and increase the frequency of extreme weather events that threaten water supplies and urban infrastructure [32–34]. Even at the current level of service, which leaves many residents without a reliable convenient water supply or a sewer connection, many large Asian cities including Chennai, Delhi, Lahore, and Dhaka are unsustainably drawing substantially more groundwater from nearby aquifers than is being replenished [35–38]. Because available water supplies are limited, as populations grow, per capita water consumption will decrease [32, 39]. This trend conflicts with aspirations for municipal systems to provide abundant water on demand in every resident’s home 24 hours a day, 7 days a week.

Thus, although there is a compelling public health case for upgrading drinking water supplies across low-income country cities, costs, political disincentives for such investments, and physical limitations on the amount of available water mean that suboptimal water distribution will continue to support ongoing transmission of typhoidal *Salmonellae* especially in low-income urban communities for the coming years.

## HOUSEHOLD-LEVEL INTERVENTIONS

Because of the difficulty in public provision of safe drinking water, several groups have advocated that households take their own steps to disinfect their drinking water [40]. These approaches include chlorine disinfection, filters, sunlight, and ultraviolet light. Although trials of the impact of household water treatment on enteric fever have not been reported, in randomized controlled trials, households given household water treatment technology and supplies have drinking water less contaminated with human fecal organisms and report less child diarrhea compared with controls [41]. The main drawback to household-level water treatment is that it shifts the responsibility of water treatment on to the households with the least capacity to implement. Indeed, fewer than 10% of African households in the bottom 40% of the income distribution report treating their drinking water with a microbiologically effective approach [42]. Even with intense behavior change, promotion, and subsidizing water treatment products, only a small minority of at-risk households consistently implement water treatment [43].

## THREAT OF WORSENING ANTIMICROBIAL RESISTANCE

In the pre-antibiotic era, in one hospital in Indonesia, 26% of patients hospitalized with blood culture-confirmed typhoid fever died [44]. The typhoidal *Salmonellae* often killed previously healthy people in the prime of their life. Resistance to available antibiotics is increasingly common among strains of typhoidal *Salmonella* [45–47]. Indeed, we face the imminent threat of the emergence of strains of *S* Typhi and *S* Paratyphi that are resistant to all available antimicrobials and thus a scenario of markedly increasing fatalities from the typhoidal *Salmonellae*. Resistant strains are spreading much more rapidly than either the development of new antimicrobial drugs [48, 49] or the improvement in water and sanitary infrastructure across low-income communities.

In the face of worsening antimicrobial resistance, effective development and deployment of vaccines against the typhoidal *Salmonellae* are a critical intermediate strategy. Even older vaccines provide a substantial level of protection [50]. Newer conjugate vaccines against *S* Typhi are expected to provide protection to a larger proportion of people who receive vaccine and provide protection that is substantially longer lasting than the 2 to 3 years of older vaccines and so could become an important element of an ongoing public health strategy to prevent enteric fever [51].

## ENGINEERING SYSTEMS TO INTERRUPT WATERBORNE TRANSMISSION

Nevertheless, vaccines are an incomplete strategy. The approach that eliminated typhoid fever as a major public health problem from Europe and North America did not depend on vaccine. Even the newer conjugate vaccine recently endorsed by the World Health Organization does not protect against *S* Paratyphi (or against other waterborne diseases including hepatitis E, hepatitis A, cholera, or cryptosporidiosis). The optimal long-term solution requires separating the human fecal stream from drinking water in communities globally.

The last 150-year history of global urban development provides compelling evidence that the capital, energy, and water-intensive 19th century approaches used by high-income water-rich countries to separate drinking water from human feces will not inevitably diffuse to low-income informal settlements. Although continued economic growth is projected to provide additional resources, with decreasing per capita water availability, resource-intensive systems that mix feces with water and then pump this slurry to a distant treatment plant may be an impractical model for impoverished urban slums. Because a ready solution is unavailable to this worsening situation, research to develop solutions should be a high priority. Such research should aim to develop new methods of supplying potable water that are effective within the severe constraints of decreasing per capita water availability, low income, and limited institutional capacity for governance. A number of groups have experimented with decentralized drinking water and wastewater treatment systems that require less energy and water [52–55], but we do not yet have a suite of approaches that are effective, affordable, and ready to be deployed across the range of communities at need.

## GOVERNANCE REQUIREMENTS FOR SAFE WATER

Providing safe drinking water to all residents of a city requires both a robust engineered system and the institutional support to finance, manage, operate, and maintain the system. Formal assessments of municipal water services in cities in low-income countries generally find these institutions are unable to retain talented technical experts, have insufficient budget for maintenance with limited capacity to raise their own revenue, little incentive to provide high-quality service or to extend water services to the poor, and routine corrupt practices that undermine equitable cost-effective service provision [24, 27, 56]. In contrast to a vaccine prevention strategy that is accomplished at a national level with a single policy decision that is then rolled out nationally, water delivery and waste management are usually local government responsibilities. Improving both the technology and the management of water and sanitary services requires specific financing and intense activities in each affected city.

Providing potable drinking water to urban communities also requires a sufficient supply of source water. Source water that is clean enough to be used for drinking requires that discharge from upstream communities [57] and watersheds more generally are appropriately managed to prevent excess pollution [58]. If watersheds are not protected, then surface water coming to cities is highly contaminated with sediments, sewage, agricultural chemicals, and other pollutants. These contaminants markedly increase the technical difficulty and costs to providing clean water. Attention to and investments in watersheds can yield major improvements in urban water quality. Modeling estimates suggest that in 25% of the world’s largest cities, investments to improve the watershed would completely pay for themselves in reduced costs of water treatment in urban communities [58]. However, watershed improvements are difficult for municipal authorities to influence, because political authority is not well aligned with watersheds [59].

## DONORS CONTRIBUTION TO TYPHOID CONTROL

It is much easier for an international donor to work with a small group of national experts in immunization to add an additional vaccine into an already functioning national system than it is to provide support to transform engineering, financial, and institutional processes of water providers in each city throughout a country. A vaccine is a simple, robust, and low maintenance intervention compared with drinking water supply and wastewater treatment, which require ongoing management and maintenance. Although most vaccines are overseen by national health authorities and delivered nationally, typhoid vaccine, in the limited instances of routine use, has most commonly been implemented subnationally. Vietnam has targeted children in high-burden districts to receive Vi polysaccharide vaccine [60]. In China, the city of Guilin has introduced Vi vaccine [60]. In India, the Delhi capital area offers routine vaccination [60], and the Navi Mumbai Municipal Corporation plans to implement a typhoid conjugate vaccine program in 2018 [61].

This subnational, indeed most commonly municipal level, implementation may reflect the heterogeneous spatial distribution of typhoid, and the assessment of political authorities that control measures in high-incidence areas is politically viable and cost-effective, but this calculus may not hold nationally. If municipal authorities continue to be the primary decision makers regarding steps to control typhoid, then public health advocates will need to work with municipal authorities to provide technical support for immunization decision making and delivery. Because immunization is a sound but incomplete solution, it would be prudent if technical support to municipal decision makers also addressed progressive steps to improve municipal water supply and sanitation, to help municipal authorities develop sound, long-term plans.

## CONCLUSIONS

Typhoid fever continues to kill thousands of people each week. With worsening antimicrobial resistance, this mortality burden could suddenly increase. Reducing typhoid and other waterborne diseases in urban settings requires focused effort in neighborhoods with compromised water delivery systems. We should shift our perspective. Instead of waiting for the 19th century approaches developed in wealthy, water-rich cities to reach high-need low-resource communities, we should prioritize developing innovative 21st century technology and management approaches to this complex unsolved problem. Generating, evaluating, and improving innovative approaches requires investing in research. Most of the needed research is not the sort of reductionist biomedical research that captures the imagination of microbiologists, but rather is a research agenda that considers the broad complex system that provides limited, unsafe, expensive drinking water to poor households. This research agenda requires understanding available surface water, groundwater, population growth, and climate change and how these issues impact each city. The research should clarify the political decisions, power dynamics, and actor incentives that affect the engineered water system scope and performance. The research should develop and evaluate robust innovative strategies to support operation, maintenance, and repair of the engineered water system in setting with severely compromised governance. Research should ultimately include developing approaches to collect the community’s fecal stream, treat it, and return it safely to the environment.

Even in the most optimistic scenario with substantial commitment to research, improvements in water and sanitation will not come quickly enough to avert the risk of highly antimicrobial resistant strains of typhoidal *Salmonella* in vulnerable urban communities. We need aggressive public health efforts that combine vaccine development and deployment with realistic planning and commitment to improving water and sanitation services in impoverished communities.

## Notes

***Financial support.*** The author’s time was supported by Stanford’s Woods Institute for the Environment and Stanford’s Center for Innovation in Global Health. The cost of publication was covered by the Bill and Melinda Gates Foundation.

***Supplement sponsorship.*** This article is part of the supplement “Surveillance for Enteric Fever in Asia Project,” sponsored by the Sabin Vaccine Institute.

***Potential conflicts of interest.*** All authors: No reported conflicts of interest. All authors have submitted the ICMJE Form for Disclosure of Potential Conflicts of Interest.

## References

[1] Sedgwick WT, MacNutt JS On the Mills-Reincke phenomenon and Hazen’s theorem concerning the decrease in mortality from diseases other than typhoid fever following the purification of public water-supplies. J Infect Dis 1910; 7:489–564.

[2] Cutler D, Miller G The role of public health improvements in health advances: the twentieth-century United States. Demography 2005; 42:1–22. 15782893 10.1353/dem.2005.0002

[3] Melosi MV. The Sanitary City. Baltimore: The Johns Hopkins University Press; 2000.

[4] Bartone C. Water pollution in Santiago: health impacts and policy alternatives. In: Chile–managing environmental problems: economic analysis of selected issues Latin American and Caribbean region report. Report No. 13061-CH. Washington DC: World Bank 1994:39–90. http://documents.worldbank.org/curated/en/424441468743350681/pdf/multi-page.pdf. Accessed 24 June 2018.

[5] Otaki Y, Otaki M, Sakura O Water systems and urban sanitation: a historical comparison of Tokyo and Singapore. J Water Health 2007; 5:259–65. 17674574

[6] Sinha A, Sazawal S, Kumar R, et al Typhoid fever in children aged less than 5 years. Lancet 1999; 354:734–7. 10475185 10.1016/S0140-6736(98)09001-1

[7] Brooks WA, Hossain A, Goswami D, et al Bacteremic typhoid fever in children in an urban slum, Bangladesh. Emerg Infect Dis 2005; 11:326–9. 15752457 10.3201/eid1102.040422 PMC3320465

[8] Sur D, Ochiai RL, Bhattacharya SK, et al A cluster-randomized effectiveness trial of Vi typhoid vaccine in India. N Engl J Med 2009; 361:335–44. 19625715 10.1056/NEJMoa0807521

[9] Breiman RF, Cosmas L, Njuguna H, et al Population-based incidence of typhoid fever in an urban informal settlement and a rural area in Kenya: implications for typhoid vaccine use in Africa. PLoS One 2012; 7:e29119. 22276105 10.1371/journal.pone.0029119 PMC3261857

[10] Lee EJ, Schwab KJ Deficiencies in drinking water distribution systems in developing countries. J Water Health 2005; 3:109–27. 16075938

[11] Shaheed A, Orgill J, Montgomery MA, Jeuland MA, Brown J Why “improved” water sources are not always safe. Bull World Health Organ 2014; 92:283–9. 24700996 10.2471/BLT.13.119594 PMC3967570

[12] Chowdhury MA, Ahmed MF, Gaffar MA Management of nonrevenue water in four cities of Bangladesh. J Am Water Works Assoc 2002; 94:64.

[13] McKenzie D, Ray I Urban water supply in India: status, reform options and possible lessons. Water Policy 2009; 11:442–60.

[14] Lambert AO A realistic basis for an international comparison of real losses from public water-supply systems. Water Environ J 2000; 14:186–92.

[15] van den Berg C Drivers of non-revenue water: a cross-national analysis. Utilities Policy 2015; 36:71–8.

[16] Frauendorfer R, Liemberger R The Issues and Challenges of Reducing Non-revenue Water. Manila: Asian Development Bank; 2010.

[17] Kumpel E, Nelson KL Mechanisms affecting water quality in an intermittent piped water supply. Environ Sci Technol 2014; 48:2766–75. 24459990 10.1021/es405054u

[18] Kumpel E, Nelson KL Comparing microbial water quality in an intermittent and continuous piped water supply. Water Res 2013; 47:5176–88. 23866140 10.1016/j.watres.2013.05.058

[19] Peal A, Evans B, Blackett I, Hawkins P, Heymans C Fecal sludge management: a comparative analysis of 12 cities. J Water Sanit Hyg Dev 2014; 4:563–75.

[20] Ercumen A, Gruber JS, Colford JM Jr Water distribution system deficiencies and gastrointestinal illness: a systematic review and meta-analysis. Environ Health Perspect 2014; 122:651–60. 24659576 10.1289/ehp.1306912 PMC4080524

[21] Karkey A, Jombart T, Walker AW, et al The ecological dynamics of fecal contamination and *Salmonella Typhi* and *Salmonella Paratyphi A* in municipal Kathmandu drinking water. PLoS Negl Trop Dis 2016; 10:e0004346. 26735696 10.1371/journal.pntd.0004346 PMC4703202

[22] Baker S, Holt KE, Clements AC, et al Combined high-resolution genotyping and geospatial analysis reveals modes of endemic urban typhoid fever transmission. Open Biol 2011; 1:110008. 22645647 10.1098/rsob.110008 PMC3352080

[23] Ercumen A, Arnold BF, Kumpel E, et al Upgrading a piped water supply from intermittent to continuous delivery and association with waterborne illness: a matched cohort study in urban India. PLoS Med 2015; 12:e1001892. 26505897 10.1371/journal.pmed.1001892 PMC4624240

[24] McIntosh AC Asian Water Supplies Reaching the Urban Poor. London: Asian Development Bank and International Water Association, 2003 https://www.adb.org/sites/default/files/publication/29250/asian-water-supplies.pdf. Accessed 24 June 2018.

[25] Segerfeldt F. Water for Sale: How Business and the Market Can Resolve the World’s Water Crisis. Washington, DC: Cato Institute; 2005.

[26] Swyngedouw EA The contradictions of urban water provision: a study of Guayaquil, Ecuador. Third World Planning Review 1995; 17:387.

[27] Davis J Corruption in public service delivery: experience from South Asia’s water and sanitation sector. World Development 2004; 32:53–71.

[28] Hackenbroch K, Hossain S “The organised encroachment of the powerful”—Everyday practices of public space and water supply in Dhaka, Bangladesh. Planning Theory & Practice 2012; 13:397–420.

[29] Hossain S The informal practice of appropriation and social control–experience from a bosti in Dhaka. Environ Urban 2013; 25:209–24.

[30] UN Habitat. Stateof the World’s Cities 2012/2013: Prosperity of Cities. London: Routledge; 2013.

[31] UN Habitat, UN ESCAP. The State of Asian and Pacific Cities 2015. London: UN-Habitat, 2015 http://www.unescap.org/sites/default/files/The%20State%20of%20Asian%20and%20Pacific%20Cities%202015.pdf. Accessed 24 June 2018.

[32] Vörösmarty CJ, McIntyre PB, Gessner MO, et al Global threats to human water security and river biodiversity. Nature 2010; 467:555–61. 20882010 10.1038/nature09440

[33] Palmer MA, Reidy Liermann CA, Nilsson C, et al Climate change and the world’s river basins: anticipating management options. Front Ecol Environ 2008; 6:81–9.

[34] Muller M Adapting to climate change water management for urban resilience. Environ Urban 2007; 19:99–113.

[35] Rodell M, Velicogna I, Famiglietti JS Satellite-based estimates of groundwater depletion in India. Nature 2009; 460:999–1002. 19675570 10.1038/nature08238

[36] Srinivasan V, Seto KC, Emerson R, Gorelick SM The impact of urbanization on water vulnerability: a coupled human–environment system approach for Chennai, India. Global Environ Chang 2013; 23:229–39.

[37] Briscoe J, Qamar U. Pakistan’s Water Economy: Running Dry. Karachi: Oxford University Press World Bank document; 2006.

[38] Hoque MA, Hoque MM, Ahmed KM Declining groundwater level and aquifer dewatering in Dhaka metropolitan area, Bangladesh: causes and quantification. Hydrogeol J 2007; 15:1523–34.

[39] World Economic Forum Water Initiative. Water Security: The Water-Food-Energy-Climate Nexus. Washington DC: Island Press; 2011.

[40] Lantagne DS, Quick R, Mintz ED. Household Water Treatment and Safe: Storage Options in Developing Countries. Water Stories: Expanding Opportunities in Small Scale Water and Sanitation Projects. Washington DC: Woodrow Wilson International Center for Scholars; 2006.

[41] Clasen TF, Alexander KT, Sinclair D, et al Interventions to improve water quality for preventing diarrhoea. Cochrane Database Syst Rev 2015; 20:CD004794. doi:10.1002/14651858.CD004794.pub3. 10.1002/14651858.CD004794.pub3 PMC4625648 26488938

[42] Rosa G, Clasen T Estimating the scope of household water treatment in low- and medium-income countries. Am J Trop Med Hyg 2010; 82:289–300. 20134007 10.4269/ajtmh.2010.09-0382 PMC2813171

[43] Stockman LJ, Fischer TK, Deming M, et al Point-of-use water treatment and use among mothers in Malawi. Emerg Infect Dis 2007; 13:1077–80. 18214185 10.3201/eid1307.070767 PMC2878214

[44] van den Bergh ET, Gasem MH, Keuter M, Dolmans MV Outcome in three groups of patients with typhoid fever in Indonesia between 1948 and 1990. Trop Med Int Health 1999; 4:211–5. 10223217 10.1046/j.1365-3156.1999.43374.x

[45] Rahman BA, Wasfy MO, Maksoud MA, Hanna N, Dueger E, House B Multi-drug resistance and reduced susceptibility to ciprofloxacin among *Salmonella enterica* serovar Typhi isolates from the Middle East and Central Asia. New Microbes New Infect 2014; 2:88–92. 25356352 10.1002/nmi2.46 PMC4184576

[46] Jain S, Das Chugh T Antimicrobial resistance among blood culture isolates of *Salmonella enterica* in New Delhi. J Infect Dev Ctries 2013; 7:788–95. 24240035 10.3855/jidc.3030

[47] Saha SK, Talukder SY, Islam M, Saha S A highly ceftriaxone-resistant *Salmonella* typhi in Bangladesh. Pediatr Infect Dis J 1999; 18:387. 10223698 10.1097/00006454-199904000-00018

[48] Spellberg B, Powers JH, Brass EP, Miller LG, Edwards JE Jr Trends in antimicrobial drug development: implications for the future. Clin Infect Dis 2004; 38:1279–86. 15127341 10.1086/420937

[49] Norrby SR, Nord CE, Finch R Lack of development of new antimicrobial drugs: a potential serious threat to public health. Lancet Infect Dis 2005; 5:115–9. 15680781 10.1016/S1473-3099(05)01283-1

[50] Anwar E, Goldberg E, Fraser A, Acosta CJ, Paul M, Leibovici L Vaccines for preventing typhoid fever. Cochrane Database Syst Rev 2014; 1:CD001261. 10.1002/14651858.CD001261.pub3 24385413

[51] Szu SC Development of Vi conjugate - a new generation of typhoid vaccine. Expert Rev Vaccines 2013; 12:1273–86. 24156285 10.1586/14760584.2013.845529

[52] Parkinson J, Tayler K Decentralized wastewater management in peri-urban areas in low-income countries. Environ Urban 2003; 15:75–90.

[53] Pickering AJ, Crider Y, Amin N, et al Differences in field effectiveness and adoption between a novel automated chlorination system and household manual chlorination of drinking water in Dhaka, Bangladesh: a randomized controlled trial. PLoS One 2015; 10:e0118397. 25734448 10.1371/journal.pone.0118397 PMC4348460

[54] Massoud MA, Tarhini A, Nasr JA Decentralized approaches to wastewater treatment and management: applicability in developing countries. J Environ Manage 2009; 90:652–9. 18701206 10.1016/j.jenvman.2008.07.001

[55] Winblad U. Towards an EcologicalApproach to Sanitation. Stockholm: Swedish International Development Cooperation Agency (Sida), 1997.

[56] González-Gómez F, García-Rubio MA, Guardiola J Why is non-revenue water so high in so many cities?Int J Water Res D 2011; 27:345–60.

[57] Beach B, Ferrie J, Saavedra M, Troesken W Typhoid fever, water quality, and human capital formation. J Econ Hist 2016; 76:41–75.

[58] McDonald RI, Shemie D. Urban Water Blueprint: Mapping Conservation Solutions to the Global Water Challenge. Washington, DC: The Nature Conservancy; 2014.

[59] Blomquist W, Schlager E Political pitfalls of integrated watershed management. Soc Nat Resour 2005; 18:101–17.

[60] Khan MI, Ochiai RL, Clemens JD Population impact of Vi capsular polysaccharide vaccine. Expert Rev Vaccines 2010; 9:485–96. 20450323 10.1586/erv.10.43

[61] World Health Organization. Informal Consultation of Experts on Typhoid Fever, South East Asia Region. Delhi: World Health Organization, Regional Office for South-East Asia; 2016.

